# Anti-tumor Activity of* Ferulago angulata* Boiss. Extract in Gastric Cancer Cell Line via Induction of Apoptosis

**Published:** 2014

**Authors:** Shafagh Heidari, Hassan Akrami, Roghaye Gharaei, Ali Jalili, Hamid Mahdiuni, Elham Golezar

**Affiliations:** a *Department of Biology, Faculty of Science, Razi University, Kermanshah, Iran.*; b *Cellular and Molecular Research Center, Kurdistan University of Medical Sciences, Sanandaj, Kurdistan, Iran. *

**Keywords:** Apoptosis, Antioxidant activity, Cytotoxicity, *Ferulago angulata* Boiss*.*, Human adenocarcinoma gastric cell line (AGS)

## Abstract

*Ferulago angulata* Boiss. known in Iran as Chavir, has some bioactive compounds having antioxidant activity. Because of its antioxidant activities, it sounded Chavir extract can be a good candidate for finding chemopreventive agents having inductive apoptosis properties on cancer cells. In this study, the cytotoxic effects and proapoptotic activities of Chavir’s leaf and flower extracts were investigated on human adenocarcinoma gastric cell line (AGS). The ferric reducing antioxidant power (FRAP) assay was used to determine antioxidant activity of the extract. Cytotoxic effects of the extract were performed by trypan blue and neutral red assays. For apoptosis detection, we used Annexin V staining, flow cytometry and DNA fragmentation assays. The FRAP assay results showed that antioxidant activity of leaf extract was higher than flower extract. Cytotoxicity and apoptosis–inducing activity of flower and leaf extracts changed coordinately, indicating the cytotoxicity of chavir extracts is due probably to induce apoptosis. Our results revealed that the cytotoxic effects of *F. angulate* Boiss*.* extracts on AGS cell line is close to some other plant extracts such as *Rhus verniciflua Stokes *(RVS) and *Scutellaria litwinowii*. This is the first study on cytotoxic and apoptosis–inducing effects of chavir leaf and flower extracts against AGS cell line. The Further investigation can be identification of the agent(s) by which these effects is observed.

## Introduction

Cancer is one of the leading causes of death, more than seven million in year, worldwide. Gastric cancer is the second most common cause of cancer-related death (700,000 deaths) annually. Almost two-thirds of the cases occur in developing countries (1). Currently, there are some inefficient treatments for cancer, including surgery, radiotherapy and chemotherapy; therefore, searching to find new effective therapies and anti-cancer drugs is one of the most important aims in medicine and pharmacology, respectively (2-4).

In cancer cells, cell cycle control become deregulated, and cells have an imbalanced proliferation to apoptosis ratio. Resistance to apoptosis is one of the hallmarks of human cancers, thus induction of apoptosis in malignant cells is a popular **strategy** for **cancer therapy.** Apoptotic cells have some typical features, including cell shrinkage, plasma membrane blebbing, phosphatidylserine flip-flop, chromatin condensation and DNA fragmentation (5-7).

In recent years, due to relatively inexpensive and nontoxic properties of phytochemicals or chemopreventive agents with anti-cancer effects, it is interested in herbal medicine increased, and more than 3000 plant species have been used to treat cancer (2,8). Some chemopreventive agents in combination with chemotherapeutic agents can enhance their anti-cancer effects at the lower doses and reduce chemotherapy-induced toxicity. In this regard, the first agents to advance into clinical use were the so-called vinca alkaloids, vinblastine (VLB) and vincristine (VCR). VLB is used for the treatment of leukemias, lymphomas, advanced testicular cancer, breast and lung cancers, and VCR, in addition to the treatment of lymphomas, also shows efficacy against leukemias (2, 9). In addition, an increasing number of inducible apoptotic chemopreventive agents (*e.g*., certain retinoids, nonsteroidal anti-inflammatory drugs, polyphenols, and vanilloids) have been shown to block the tumor cells proliferation in malignant cells *in-vitro *or *in-vivo *(10).

*F. angulata* Boiss. known as Chavir in Iran, is a perennial shrub 60-150 cm tall, growing at altitudes of 1900-3200 m above sea level, yellow colored flowers and thin compound leaves (11-13), and it is distributed in Iran, Turkey, Iraq, Greece, Serbia and Macedonia (14,15). Seven out of thirty-five species of genus *Ferulago*, a member of the Apiaceae plant family, are found in Iran (16,17). Chavir has been used traditionally as remedy for dyspepsia (digestive pains), moreover, a report in Iran showed it also has antimicrobial activity (18). To our knowledge, the major constituents of *F. angulata *Boiss. essential oil are composed of cis–ocimene, α- pinene, boronyl acetate and Germacren-D (11,19).

Natural antioxidants present in plants have attracted interest because of their abilities to scavenge free radicals, which are reactive molecules with unpaired electrons that are able to damage vast varieties of cellular macromolecules including proteins, DNA and lipid bilayer membranes. Reactive oxygen species (ROS) are linked to some diseases such as cancer. Natural antioxidants presented in plants have attracted interest because of their abilities to scavenge these active species (20). Due to the potential of antioxidants to decrease the risk of the cancer, we analyzed antioxidant capacity of alcoholic extracts of aerial parts of *F. angulata* Boiss., by ferric reducing antioxidant power (FRAP) assay.

The aim of our study was to evaluate the cytotoxicity power and apoptosis-inducing effects of *F. angulata* Boiss. ethanol extracts on gastric adenocarcinoma cell line (AGS) in order to determine its probable anti-cancer properties.

## Experimental

*Chemicals and reagents*

Trypan blue (Sigma–Aldrich, USA), Neutral red (Sigma–Aldrich, USA), EDTA (Merck, Germany), Trypsin (Sigma–Aldrich, USA), Roswell Park Memorial Institute medium (RPMI) 1640 (Sigma–Aldrich, USA), Fetal Bovine Serum (FBS) (Biocrom, AG), Penicillin G (Sigma–Aldrich, USA), Streptomycin Sulfate (Sigma–Aldrich, USA), DMSO (Merck, Germany), Ethidium bromide (Merck, Germany), GeneRuler™ 1 kb DNA Ladder (Fermentas, China), Annexin V-FLUOS staining kit (Roche, Germany), Quick apoptotic DNA ladder detection kit (Abcam, USA). Trolox (Sigma–Aldrich, USA), TPTZ (Tripyridyl-s-Triazine), Iron III-clorure-6-hydrate (FeCl_3_.6H_2_O), Sodium acetate and all of the solvents used in the present work were purchased from Merck (Germany).

*Sample preparation and extraction procedure*

*F. angulata *Boiss. was collected from the Shahoo Mountain– Kermanshah province (west of Iran) in May 2010 and authenticated by Dr. Seyedmohammad Masoumi, assistant professor of botany, Razi University, Kermanshah, Iran (Voucher Number: 580). Alcoholic extraction of aerial parts (flower and leaf) of *F. angulata *Boiss. was done as follow. After harvesting, aerial parts of the plant were dried at room temperature 20–25 C and kept in dark for 7 days. The yield of dried flower and leaf was about 7% (w/w) of the starting dried flower and leaf. Then, approximately 50 g of grounded dried flowers and leaves were put in a Soxhlet apparatus separately and were mixed with ether de petrole (~3 times) until it became colorless. Then, the two almost resin-free suspensions were centrifuged (at 3000 *g* for 10 min); the resulting pellets were re-mixed with 500 mL ethanol and were stirred for 4 days. After centrifugation (at 3000 g for 15 min), the supernatants were evaporated under vacuum to dry and turn into powders. 

All extracts were dissolved in DMSO and subsequently diluted in RPMI 1640 containing 10% FBS at desired concentrations in the range of 20–240 μg/mL. The DMSO concentration in the culture medium did not exceed 1% v/v, which had no cytotoxic effect on AGS cell line.

*FRAP assay*

The FRAP assay was performed according to Benzie and Strain (21), with some modifications. The fresh working solution (FRAP solution) was prepared by mixing 25 mL acetate buffer (300 mM, pH 3.6), 2.5 mL TPTZ solution (10 mM in 40 mM HCl), and 2.5 mL FeCl_3_.6H_2_O solution (20 mM) and then warmed at 37 C before using. Flower or leaf extracts (10 μL) were allowed to react with 990 μL of the FRAP solution for 30 min in the dark condition. Readings of the colored product were then taken at 595 nm using a Carry MODEL spectrophotometer. Results are expressed in μM Trolox (TE)/ g dry mass of samples.

*Cell culture*

AGS, a gastric adenocarcinoma cell line, was provided from National Cell Bank of Iran (NCBI), Pasteur Institute, Tehran, Iran. The cells were grown as a monolayer culture in RPMI 1640 medium, supplemented with 10% (v/v) heat-inactivated FBS, 100 U/mL penicillin G and 100 μg/mL streptomycin, incubated at 37 °C in a humidified incubator containing 5% CO_2_ atmosphere. The culture media were changed twice a week.

*Cell viability assays*

*a) Trypan blue exclusion assay*

AGS cells (1 × 10^5 ^cells/well) were cultured on 24–well plates in RPMI 1640 containing 10% FBS and supplemented with 100 U/mL penicillin and 100 μg/mL streptomycin. After 24 h, AGS cells (reached around 60–70% confluent) were treated with the different concentrations of *F. angulata *Boiss. extracts (20, 40, 60, 80, 100, 120, 140, 160 µg/mL) for 24 h and 48 h. The control groups included untreated AGS cells and 1% DMSO-treated AGS cells. At the end of incubation period, cell viability was measured by trypan blue dye using a hemocytometer. The percentage of cytotoxicity was expressed as 100% minus the cell viability percentage**.**

*b) Neutral red assay*

The neutral red assay was carried out according the method described by Borenfreund and Puerner (22). AGS cells (1 × 10^5 ^cells/well) were cultured on 24–well plates in triplicate and incubated for 24 h in RPMI 1640 containing appropriate antibiotics and supplemented with 10% FBS in a humidified atmosphere of 5% CO_2_ at 37 °C. Then, the medium of each well of 24–well plates was replaced by fresh RPMI 1640 containing 3% FBS, supplemented with appropriate antibiotics and conducted using several concentrations of *F. angulata *Boiss. extracts (20, 40, 60, 80, 100, 120, 140, 160) µg/mL in a humidified atmosphere of 5% CO_2_ at 37 °C for 24 h and 48 h.

At the end of incubation period, AGS cells were washed by PBS, and then incubated with neutral red dye for 2 h in CO_2 _incubator to promote the uptake of the dye by cells. Subsequently, the supernatant was removed and the cells were washed by warmed PBS. Then one mL/well of extraction solution (H_2_O: acetic acid: ethanol) (49:1:50) was added followed by incubation in the dark shaking incubator at 37 ˚C for 15 min. The absorbance of solutions was read with the spectrophotometer at 540 nm wavelength using extraction solution as a reference. The percentage of cytotoxicity was determined by comparing optical density (OD) of treated AGS cells with untreated AGS cells as control in 540 nm.

Also, the 50% inhibitory concentration (IC_50_) values of extracts against AGS cells were determined from dose response curves.

*Annexin V-PI double staining assay*

To observe cell surface exposure of phosphatidylserine, an early marker of apoptosis, AGS cells (1 × 10^5 ^cells /well) were seeded on 24–well plates in a humidified atmosphere of 5% CO_2_ at 37 °C for 24 h before the treatment. After that, *F. angulata *Boiss. extracts at concentrations of 80 μg/mL and 160 μg/mL were added to each well in a humidified atmosphere of 5% CO_2_ at 37 °C. After 24 h incubation, cells were washed with PBS and stained with Annexin V–FLUOS and PI according to the protocol of Annexin V–FLUOS staining kit. Subsequently, the cells were examined under a fluorescence microscope at the range of (515–565) nm. Annexin V (Green fluorescence) uptake was observed in early apoptotic cells and red fluorescence was observed in necrotic cells or late apoptotic cells which uptake the PI dye.

*Flow cytometry* *analysis*

The apoptotic rate was detected by flow cytometry. AGS cells (1 × 10^6^) cells /well were plated in 6–well plates for 24 h and then treated with *F. angulata *Boiss. at concentrations of 80 μg/mL and 160 μg/mL for an additional 24 h in a 5% CO_2_ humidified atmosphere at 37 °C. At the end of incubation period, the treated AGS cells and controls were harvested and incubated with Annexin V and PI for 15 min before being analyzed on flow cytometer with 488 nm excitation and 515 nm for Annexin V detection and a filter with the wavelengths above 600 nm for PI detection. 

*DNA fragmentation assay*

AGS cells (1 **×** 10^6^ cells/well) were treated with different concentrations of *F. angulata *Boiss. leaf extracts (80, 120, 160 and 200) µg/mL and flower extracts (120, 160, 200 and 240) µg/mL at 37 °C in a humidified atmosphere of 5% CO_2_ for 24 h. DNA fragmentation analysis was performed according to the manufacturer’s protocol. Fragmented DNA was separated on 1% agarose gel. 

*Statistical analysis*

Results were presented as mean ± standard error of the mean (S.E.M.) of values obtained by at least triplicate measurements from three independent experiments. Student’s t-test was applied to evaluate the differences between treated and control groups (two-group comparison). Two-way ANOVA was used for multiple comparisons. p*-*values less than 0.05 were considered as statistically significant (*p* <0.05). Statistical analysis of data was performed with SPSS (Version 16: SPSS. Link. USA). IC_50_ (the 50% inhibitory concentration) values were estimated using linear regression analysis of plots of the percent of cytotoxic activity against the concentration of the samples using Microsoft Excel Software Program.

## Results

*The antioxidant activity of F. angulata Boiss.*

The FRAP assay was originally applied to measure the ferric reducing power of plasma (21), but has been extended to other samples such as plant extracts (23), juices (24), etc. When a ferric- tripyridyltriazine (FeIII-TPTZ) (TPTZ: 2,4,6-tripyridyls- triazine) complex is reduced to the ferrous (FeII) form, developed an intense blue color with a maximum absorption at 595 nm.

The FRAP values of leaf extract and flower extract were 185.4 ± 16.8 µM TE/g dry mass and 98.7 ± 1.8 µM TE/g dry mass of the plant samples, respectively. The values of the alcoholic extracts of aerial parts of *F. angulata* Boiss. showed that antiradical activity of leaf extract was stronger than of flower extract (*p *<0.05).

*Effect of F. angulata *Boiss.* leaf and flower extracts on AGS Cell viability*

**Figure 1:**
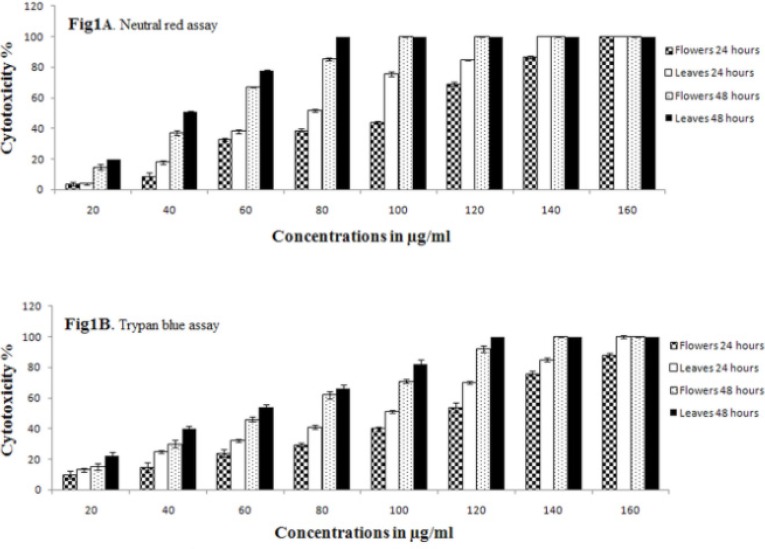
Cytotoxic effect of various concentrations (20, 40, 60, 80, 100, 120, 140 and 160) μg/mL of *F. angulata *Boiss. flower and leaf extracts on AGS cells at 24 h and 48 h. A) The percentage of cytotoxicity values were measured with neutral red and B) The percentage of cytotoxicity values were measured with trypan blue assays. Bars are represented by the mean ± S.E.M. of three independent experiments (*n *= 3). (*p* <0.05 vs. control group).

Effect of *F. angulata *Boiss. leaf and flower extracts on cell viability of AGS cells were analyzed by trypan blue exclusion and neutral red uptake assays and IC_50_ values were calculated for these extracts against AGS cells (Table 2). Results of cell viability assays are shown in Figure 1 and Table 1. Treatment of AGS cells with leaf extracts (≥20 µg/mL) showed cytotoxicity at 24 h and 48 h (Figure 1 and Table 1). While, flower extracts showed cytotoxic effect on AGS cells at concentrations equal or above 40 µg/mL for 24 h and concentrations equal or above 20 µg/mL for 48 h, as compared to the control groups (*p*<0.05) (Figure 1 and Table 1). Our results showed that both leaf and flower extracts displayed cytotoxicity in AGS cells at various concentrations with time and in a concentration dependent manner. Altogether, the leaf extract exhibited higher cytotoxic activity than flower extract (*p*<0.05).

**Table 1:** Cytotoxic effects of different concentrations of *F. angulata *Boiss. extracts on AGS cells. Each value is represented by the mean ± S.E.M. of three independent experiments (*n *= 3) of three replicates (*P* < 0.05 vs. control group).

**A)** Trypan blue assay									
**Concentration** **μg/mL**									
**Plant part** **tested**		20	40	60	**80**	**100**	**120**	**140**	**160**
Leaves	24 h	13.0±2.08	25.0±1.52	32.0±3.05	41.0±2.64	51.0±1.52	70.0±2.08	85.0±1.52	100.0±0
	48 h	22.0±2.51	39.67±2.18	54.0±1.52	66.0±2.30	82.0±3.05	100.0±0	100.0±0	100.0±0
Flowers	24 h	10.0±2.513	14.67±2.90	24.0±2.52	29.33±1.20	40.0±1.15	54.0±3.05	76.0±1.73	88.0±1.53
	48 h	15.0±2.08	30.0±2.31	46.0±1.53	62.0±2.52	71.0±1.15	92.0±2.0	100.0±0	100.0±0
*S.E.M. = Standard error of the mean.*									
**B)** Neutral red assay									
**Concentration μg/mL**									
** Plant part** **tested**		**20**	**40**	**60**	**80**	**100**	**120**	**140**	**160**
Leaves	24 h	3.85±0.63	18.05±1.25	38.05±1.47	51.67±0.90	75.62±1.79	84.91±0.41	100.0±0	100.0±0
	48 h	19.45±0.26	50.87±0.58	77.80±0.70	100.0±0	100.0±0	100.0±0	100.0±0	100.0±0
Flowers	24 h	3.84±1.29	8.77±2.32	33.04±0.93	38.73±0.91	49.93±0.76	69.27±1.19	86.68±0.79	100.0±0
	48 h	14.75±1.79	37.06±1.48	67.05±0.54	85.62±0.92	100.0±0	100.0±0	100.0±0	100.0±0

*S.E.M. = Standard error of the mean.*

**Table 2:** Cytotoxicity of *F. angulata *Boiss. extracts on AGS cells expressed as IC_50_ values. Results are represented by the mean ± S.E.M. of three replicates in three independent experiments (*P* < 0.05 vs. control group).

**Plant part tested**	**Extracts**	**IC**_50_ ** (µg/mL)** **24 h Trypan blue assay**	**IC**_50_ ** (µg/mL)** **48 h Trypan blue assay**	**IC**_50_ ** (μg/mL)** **24 h Neutral Red assay**	**IC**_50_ ** (μg/mL) 48 h Neutral Red assay**
Leaves	Ethanolic	82.20	60.41	76.65	61.70
Flowers	Ethanolic	101.81	74.00	89.84	63.50

*S.E.M.=standard error of the mean.*

*Apoptosis induction effect of F. angulata *Boiss.* leaf and flower extracts on AGS cells*

AGS cells treated with leaf and flower extracts further analyzed for their ability to induce apoptosis using DNA fragmentation assay and Annexin V–FLUOS staining assay. 

*a) DNA fragmentation assay*

**Figure 2:**
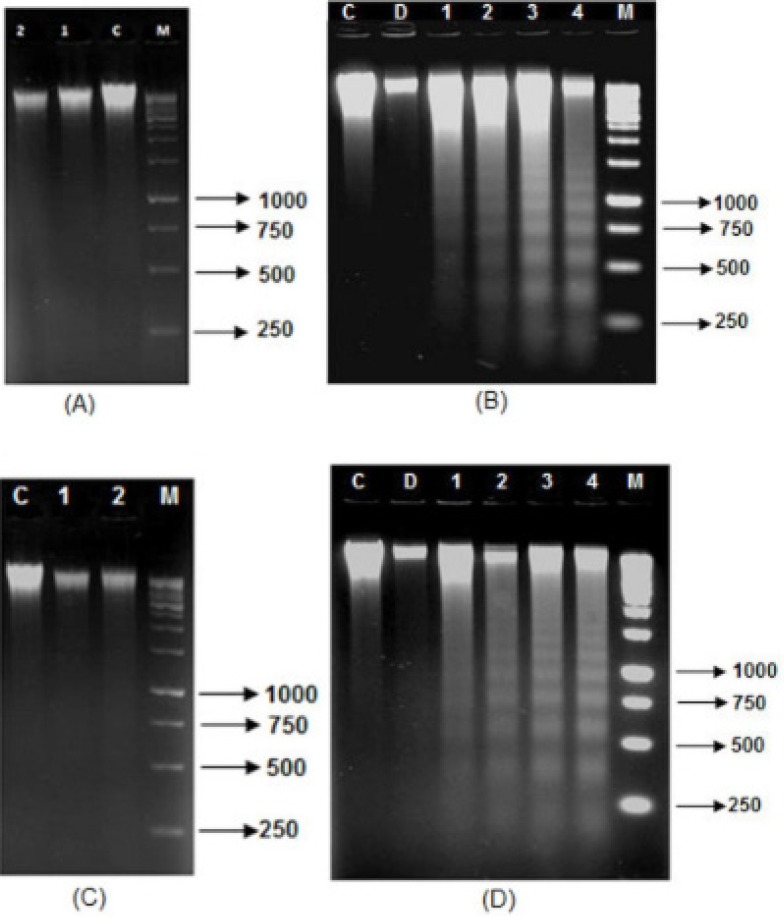
DNA fragmentation assay in AGS cells treated with different concentrations of *F. angulata *Boiss. leaf and flower extracts by 1% agarose gel electrophoresis (M stands for DNA marker, C stands for control, D stands for 1% DMSO–treated cells). A) AGS cells treated with 160 µg/mL (line 1), 200 µg/mL (line 2) leaf extracts for 24 h. B) AGS cells treated with leaf extracts for 48 h. 1 stands for 80 µg/mL. 2 stands for 120 µg/mL. 3 stands for 160 µg/mL. 4 stands for 200 µg/mL. C) AGS cells treated with 200 µg/mL (line 1), 240 µg/mL (line 2) flower extracts for 24 h. D) AGS cells treated with flower extract for 48 h. 1 stands for 120 µg/mL. 2 stands for 160 µg/mL. 3 stands for 200 µg/mL. 4 stands for 240 µg/mL.

The supernatants from AGS cells, which had been incubated with different concentrations of leaf and flower extracts including (80, 120, 160, 200 and 240) μg/mL for 24 and 48 h, were extracted for DNA and examined by agarose-gel electrophoresis (Figure 2). No DNA fragmentation was observed in AGS cells treated with leaf and flower extracts for 24 h (Figures 2A, 2C). Interestingly, DNA fragmentation was evident in AGS cells treated with (80, 120, 160 and 200) μg/mL of leaf extracts and (120, 160, 200 and 240) μg/mL of flower extracts, in a concentration dependent manner, at 48 h (Figures 2B, 2D).

*b) Annexin V–FLUOS staining assay*

**Figure 3:**
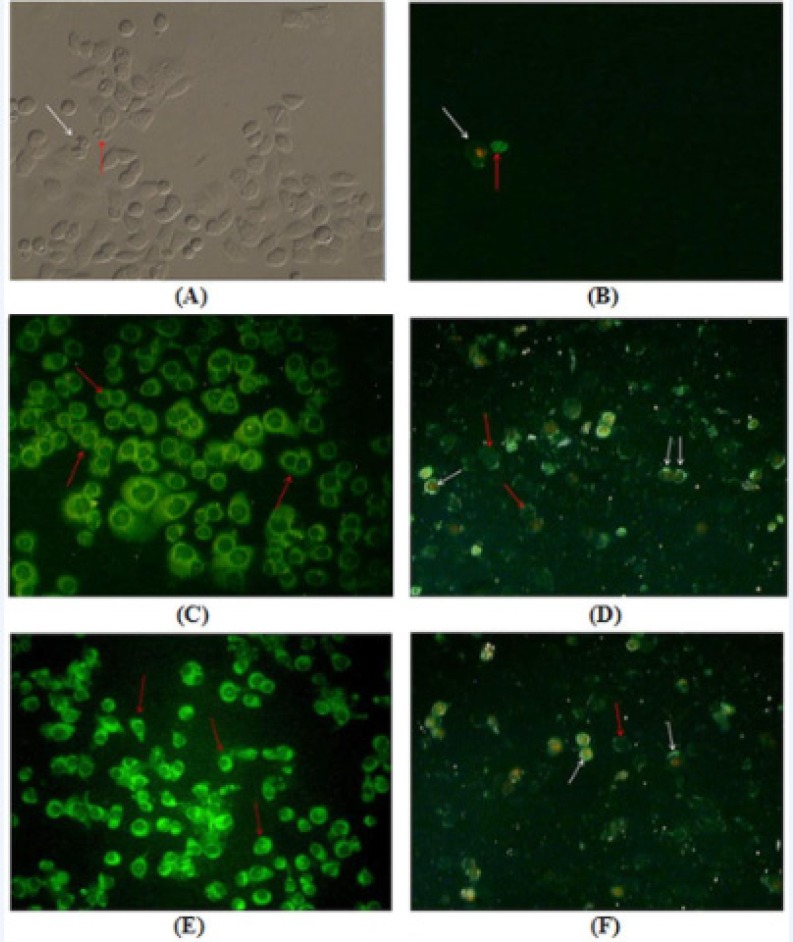
Annexin V–FLUOS and PI staining of AGS cells treated with *F. angulata *Boiss. leaf and flower extracts for 24 h. Early apoptotic cells with only Annexin V–FLUOS positive staining were recognized by green plasma membrane (red arrows), while late stage apoptotic cells with both Annexin V–FLUOS and PI positive staining were observed with green membrane and red nucleus (white arrows). A) and B) Untreated AGS cells observed under inverted microscope and fluorescence microscope, respectively. C) and D) AGS cells treated with 80 μg/mL and 160 μg/mL concentrations of* F. angulata *Boiss. leaf extracts, respectively. E) and F) AGS cells treated with 80 μg/mL and 160 μg/mL concentrations of* F. angulata *Boiss. flower extracts, respectively. Magnification 200X

**Figure 4:**
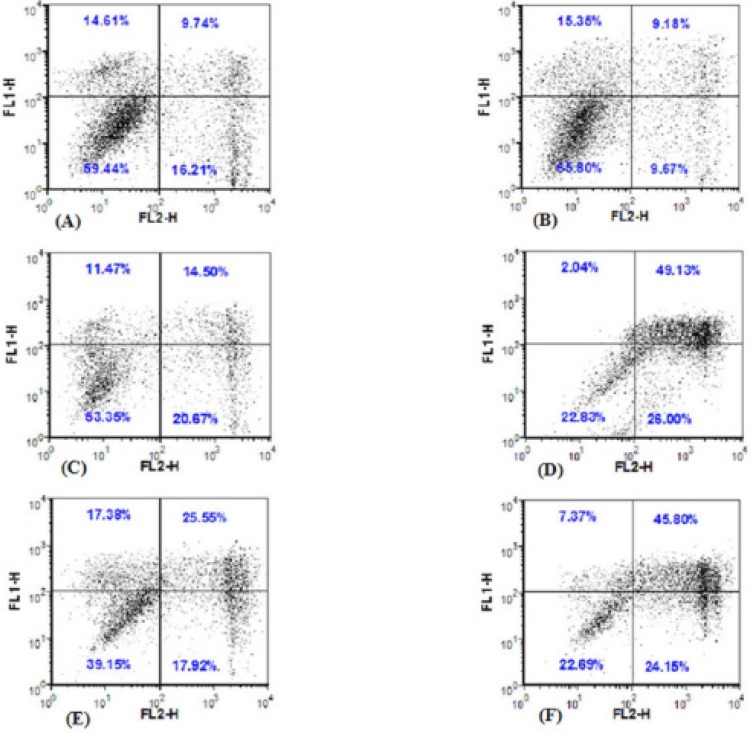
Flow cytometric analysis by Annexin V–FLUOS (FL1-H) in *x*-axis and PI (FL2-H) in *y*-axis double staining of AGS cells treated with *F. angulata *Boiss. leaf and flower extracts at 24 h. Living cell (Annexin V^−^/PI^−^) populations were located in the lower left quadrant (LL), the apoptotic cells stained by annexin V and unstained by propidium iodide in the lower right quadrant (LR), late apoptotic (Annexin V^+^/PI^+^) populations were located in the upper right quadrant (UR), and necrotic cell (Annexin V^−^/PI^+^) populations were presented in the upper left quadrant (UL). Dot plots of Annexin V/PI staining are shown in A) (untreated AGS cells), B) (1% DMSO-treated AGS cells), C) AGS cells treated with 80 μg/mL* F. angulata *leaf extracts exhibited 14.5% late stage apoptosis and 20.67% early stage apoptosis, D) AGS cells treated with 160 μg/mL of* F. angulata *Boiss. leaf extracts had 49.13% late stage apoptosis and 26% early stage apoptosis, E) AGS cells treated with 80 μg/mL* F. angulata *flower extracts showed 25.55% late stage apoptosis, 17.92% early stage apoptosis, F) AGS cells treated with 160 μg/mL* F. angulata *Boiss. flower extracts showed 45.80% late stage apoptosis, 24.15% early stage apoptosis (*P* < 0.05).

AGS cells were treated with 80 μg/mL and 160 μg/mL of leaf and flower extracts for 24 h. Apoptotic cells were observed under fluorescence microscope after staining with Annexin V–FLUOS and PI (Figure 3). Early apoptotic cells with only Annexin V positive staining were recognized by green plasma membrane, while late stage apoptotic cells with both Annexin V and PI positive staining were observed as green plasma membrane with red nucleus. Furthermore, rate of apoptosis was quantified using flow cytometry (Figure 4).

AGS cells showed 49.13% late apoptosis and 26% early apoptosis when treated with 160 μg/mL of leaf extract (Figure 4D); the cells also showed 45.8% late apoptosis and 24.15% early apoptosis when treated with 160 μg/mL of flower extract (p-values < 0.05) (Figure 4F). 

## Discussion

Due to lack of effective treatments, cancer is a fatal disease rating the top three cause of death (1). Major issues concerning most available chemotherapies are severe side effects and chemo-resistance (25,26).

Plants are a useful source of anti-cancer components (8). In many countries, the cytotoxic activities of some native plants have been studied on different cancer cell lines to discover new anti-cancer agents (20,27,28,29,30). Additionally, some medicinal plants were investigated for antitumor activity against AGS cell line, which are mentioned in the following paragraph.

Ethanol extract of* Rhus verniciflua Stokes* (RVS) caused a dose-dependent decrease in the cell viability (IC_50_: 50 μg/mL) and induced apoptosis via the intrinsic death pathway in human gastric cancer cells (31). The water extract of *Strychni Semen* (ESS) reduced AGS cells growth and induced apoptosis in a concentration-dependent manner (32). Methanol extract of *Artemisia annua* inhibited AGS gastric cancer cell growth by inducing apoptosis and inhibiting cell proliferation (IC_50_: 500 μg/mL) (33). Methanol extract of *Scutellaria litwinowii* induced apoptosis and inhibited the proliferation of AGS cells by IC_50_: 121.2 ± 3.1 μg/mL (28). Flavonioids isolated from *Citrus aurantium *L. had the anti-proliferative activity on AGS cells with IC_50_ value of 99 μg/mL and induced apoptosis by caspase activation (34).

In our study, we investigated the antioxidant effect and cytotoxic activity of the ethanol extract of *F. angulata *Boiss. on AGS cell line. To the best of our knowledge, there is a study on antioxidant activity of the essential oil of *F. angulata *Boiss. by peroxide value and thiobarbituric index (35). Here FRAP assay was used to confirm the antioxidant property of *F. angulata *Boiss. flower and leaf extracts.

Both trypan blue and neutral red assay results confirmed that the cytotoxic effects of extracts of* F. angulata *Boiss. on AGS cell line are close to ethanol extract of *Rhus verniciflua Stokes *(RVS) and *Scutellaria litwinowii*, Nevertheless, it has stronger cytotoxic effect than *Artemisia annua* extract.

During the early stage of apoptosis, phosphatidylserines in cell membrane translocate to the outer leaflet of the plasma membrane. Double staining of annexin V-FLUOS and PI can be used to detect exposed for the detection of exposed phosphatidylserine using fluorescence microscopy and flow cytometry (36). Annexin V exposes phosphatidylserine of the early and late apoptotic cells, while propidium iodide (PI) stains the nucleus of the cells, which have lost their membrane integrity in the late stage of apoptosis or necrosis (37). Therefore, the annexin V^+^/PI^-^ cells show early apoptosis and the annexin V^+^/PI^+ ^cells show late apoptosis or necrosis (38,39).

Based on annexin V/PI staining results, many annexin V^+^/PI^-^ cells were observed in the cells treated with 80 μg/mL of flower and leaf extracts, indicating the early stage of apoptosis. While, in the cells treated with 160 μg/mL flower and leaf extracts in addition to annexin V^+^/PI^-^ cells, some annexin V^+^/PI^+ ^cells were also observed, indicating some cells in the late stage of apoptosis (secondary necrosis). Furthermore, flow cytometric analysis revealed that the number of early and late apoptotic cells were significantly increased, in a concentration-dependent manner, following treatment with 80 μg/mL and 160 μg/mL of flower and leaf extracts as compared to the control groups. These results indicated that the cytotoxicity of *F. angulata *Boiss. toward AGS cells was mainly caused by its ability to induce apoptosis.

For further confirmation of the ability of *F. angulata *Boiss. flower and leaf extracts to induce apoptosis in AGS cells, additional biochemical properties were monitored. A hallmark feature of apoptosis is fragmentation of apoptotic nuclear DNA in a ladder electrophoretic pattern. Necrosis, on the other hand, is characterized by random digestion of DNA forming a “smear” on agarose gel (40,41).

In the present study, we have shown that AGS cells have a dose-dependent apoptosis-specific DNA fragmentation when they treated with *F. angulata *Boiss. flower and leaf extracts, indicating that the cytotoxic effect of *F. angulata *Boiss. flower and leaf extracts on AGS cells was induced by apoptosis; a pathway was previously reported with some other plant extracts (37,42,43,44).

## Conclusion

Based on the outcome of our study, *F. angulata *Boiss. flower and leaf extracts have an ability to induce apoptosis. To the best of our knowledge, this is the first report describing significant cytotoxic and proapoptotic effects of *F. angulata *Boiss. leaf and flower extracts against AGS cell line. Since one of the most important challenges for anti-cancer drug development is to induce apoptosis in malignant cells, *F. angulata *Boiss. extract may be considered as a potential anti-cancer agent. This is very promising, indicating that *F. angulata *Boiss. extract contains components with anti apoptotic activity; therefore, it can be a good candidate for further investigations to isolate, identify and test bioactive principles and elucidate the mechanisms by which *F. angulata *Boiss. flower and leaf extracts act on apoptotic signaling pathways. 
